# High Dose Steroids as First-Line Treatment Increased the Risk of In-Hospital Infections in Patients With Anti-NMDAR Encephalitis

**DOI:** 10.3389/fimmu.2021.774664

**Published:** 2021-12-17

**Authors:** Jierui Wang, Jingfang Lin, Minjin Wang, Zirui Meng, Dong Zhou, Jinmei Li

**Affiliations:** ^1^ Department of Neurology, West China Hospital, Sichuan University, Chengdu, China; ^2^ Department of Laboratory Medicine, West China Hospital, Sichuan University, Chengdu, China

**Keywords:** anti-N-methyl-D-aspartate receptor, autoimmune encephalitis, immunotherapy, steroids, infection

## Abstract

**Objective:**

To address the effects of high dose steroids on in-hospital infection and neurologic outcome in anti-N-methyl-D-aspartate receptor (anti-NMDAR) encephalitis patients.

**Methods:**

We retrospectively reviewed the clinical data of anti-NMDAR encephalitis patients in West China Hospital, the Third Hospital of Mianyang and Mianyang Central Hospital between October 2011 and August 2020. The development of infections, inflammatory factors, neurologic outcome at discharge and risk factors for in-hospital infection were assessed in patients with and without high dose steroid therapy before and after immunotherapy. Least absolute shrinkage and selection operator (LASSO) regression and logistic regression models were established to assess risk factors for in-hospital infection.

**Results:**

A total of 278 patients with anti-NMDAR encephalitis were included in the study. Thirty-four patients received high dose methylprednisolone (IVMP) therapy only, 84 patients received intravenous immunoglobulin (IVIG) therapy, and 160 patients received IVIG and IVMP therapy. Compared with the IVIG group, IVIG + IVMP group had a higher infection rate (64.38% vs 39.29%, *P* < 0.001), a higher incidence of noninfectious complications (76.25% vs 61.90%, *P* = 0.018) and a higher modified Rankin Scale (mRS) score at discharge from the hospital (3 vs 2, *P* < 0.001). Inflammatory indicators, including white blood cell (WBC) count, neutrophil-to-lymphocyte ratio (NLR) and systemic immune-inflammation index (SII), were higher (9.93 vs 5.65, 6.94 vs 3.47 and 1.47 vs 0.70, respectively, *P* < 0.001) in the IVIG + IVMP group than in the IVIG group. Moreover, lymphocyte-to-monocyte ratio (LMR) was lower (2.20 vs 2.54, *P* = 0.047) in the IVIG + IVMP group. The LASSO model showed that mRS score on admission, seizure, body temperature, uric acid (URIC), cerebrospinal fluid immunoglobulin G (CSF IgG), NLR and LMR were risk factors for in-hospital infection. The prediction model exhibited an area under the curve (AUC) of 0.885.

**Conclusions:**

High dose steroids therapy was significantly associated with higher in-hospital infectious complication rates and a poor short-term prognosis in relatively severe anti-NMDAR encephalitis patients. The established prediction model might be helpful to reduce the risk of in-hospital infection.

## 1 Introduction

Infection is the most common complication of anti-N-methyl-D-aspartate receptor (anti-NMDAR) encephalitis due to immunologic abnormalities, status epilepticus or disturbance of consciousness (1, 2). The most common infections are pneumonia and urinary tract infections (3). Among first-line immunotherapies, including steroids, intravenous immunoglobulin (IVIG) and plasma exchange, the use of high dose steroids is one of the most common strategies (4, 5). The effectiveness of high dose steroids was verified in large-scale studies; however, the high frequency of secondary infectious complications after high dose steroids was frequently ignored (6). The effect of these high-dose steroid regimens on the original infection or whether they will cause new infectious complications are unknown. Recent evidence has shown that the administration of high dose steroids is associated with pneumonia, which leads to a longer length of hospital stay (LOS), poorer functional outcomes, higher healthcare costs and even mortality. Previous studies suggested that steroids should be avoided in cases of clinical suspicion or microbiological confirmation of pneumonia (7).

Most reports to date are of unselected patients with anti-NMDAR encephalitis, and evidence for the safety of high dose steroids in anti-NMDAR encephalitis patients with infection is still insufficient. Generating evidence supporting the use of high dose steroids in this specific population is critical because proper management may help prevent unfavorable outcomes. Accordingly, in this study, we aimed to assess the effect of high dose steroids treatment on the incidence of in-hospital clinical infection, inflammatory parameters and short-term neurologic outcome and developed a prediction model based on easily obtained information on clinical and laboratory variables before corticosteroid therapy to identify risk factors for severe infection.

## 2 Methods

### 2.1 Study Design and Patients

In this retrospective study, we reviewed consecutively anti-NMDAR encephalitis patients who were admitted to the Department of Neurology at West China Hospital, the Third Hospital of Mianyang and Mianyang Central Hospital between October 2011 and August 2020. The patients met the anti-NMDAR encephalitis diagnostic criteria proposed by Graus et al. (8) All patients received first-line immunotherapy and underwent the whole immunotherapy in the follow-up investigation. The following patients were excluded: (1) patients with active infection within the 2 weeks before admission who met the following conditions: patients with infiltrating lesions on chest images, leukocyturia or abdominal pain; (2) patients with malignant tumors; (3) patients with severe hepatic or renal diseases; and (4) patients who could not fulfil the 3-month follow-up.

High dose steroid treatment was defined as 1000 mg/day methylprednisolone for 3-5 days *via* intravenous injection. IVIG was administered at a dosage of 0.4 g/kg/day for 5 days. Comparisons were made in patients who received IVIG treatment alone (IVIG group) and those who receive IVIG add to high dose intravenous methylprednisolone treatment (IVIG + IVMP group).

This study was approved by the Research Ethics Committee of West China Hospital of Sichuan University. Written informed consent was obtained from all participants or their direct relatives.

### 2.2 Data Collection

Two trained neurologists reviewed the clinical data, including demographic data, clinical phenotype, Glasgow Coma Scale score, complications, in-hospital stay, modified Rankin Scale (mRS) score, brain magnetic resonance imaging (MRI), electroencephalogram (EEG) and cerebrospinal fluid (CSF) examination.

Fasting blood samples were collected within before and 10 days after receiving first-line immunotherapy. We documented the following baseline data associated with infection: white blood cell (WBC) count, neutrophil count, lymphocyte count, monocyte count, erythrocyte sedimentation rate, C-reactive protein (CRP), procalcitonin, properdin factor B (PFB), CD3, CD4, CD8, CD4/CD8, etc. Systemic inflammation-based scores calculated based on these laboratory results included the neutrophil-to-lymphocyte ratio (NLR), lymphocyte-to-monocyte ratio (LMR) and systemic immune-inflammation index (SII). The SII was defined as follows: SII = neutrophil × platelet/lymphocyte. Urinalysis and urine and sputum cultures were performed. Chest computed tomography (CT) scan results were documented. Daily maximum axillary body temperature, as a physical factor directly associated with infection, was collected before and within 10 days after receiving first-line immunotherapy.

### 2.3 Outcomes Evaluated

#### 2.3.1 In-Hospital infection Occurrence After Immunotherapy

This study aimed to determine whether high dose steroid use was associated with in-hospital infection. In-hospital infection is defined as an infection not incubating at the time of hospital admission and occurring 48 hours or more after admission and criteria as follows: I) Pneumonia was diagnosed according to the clinical guideline of Chinese Thoracic Society (CTS)/Chinese Association of Chest Physicians (CACP), including newly emerging lesions or progressively infiltrating lesions on chest images combined with more than two of the following clinical symptoms of infection: (1) fever ≥ 38°C; (2) new cough, purulent sputum, or shortness of breath with or without chest pain; (3) signs of pulmonary consolidation and/or moist gales after 48 hours in hospital; and (4) peripheral WBC ≥ 10 × 10^9^/L or ≤ 4×10^9^/L. Severe pneumonia was defined as meeting one of the major criteria including invasive mechanical ventilation or septic shock with a need for vasopressors or with more than three of the following minor criteria: (1) respiratory rate ≥ 30 times/min; (2) partial arterial oxygen pressure to fraction of inspiration O2 ratio (PaO_2_/FiO_2_) ≤ 250 mmHg; (3) multiple pulmonary infiltration; (4) disturbance of consciousness; (5) Blood urea nitrogen (BUN) ≥ 7.14 mmol/L; and (6) systolic blood pressure < 90 mmHg. II) Urinary tract infections (UTIs) were defined as the presence of at least one of urinary symptoms (dysuria, urgency, frequency, flank pain, or suprapubic tenderness) and one of the positive laboratory reports: (1) urine sample positive for nitrites and/or pyuria; and (2) positive urine culture. For symptomatic women, a positive culture definition is the presence of one or two isolates at ≥ 10^2^ CFU/mL. In males, a culture of ≥10^3^ CFU/mL is considered to be significant. III) Infection was also diagnosed if the temperature was above 38.0°C for at least 2 determinations with additional WBCs but no determined focus.

#### 2.3.2 Determining the Effect of In-Hospital Infection on Clinical Outcome After Steroid Therapy Use

The LOS and mRS score at discharge from the hospital were examined in anti-NMDAR encephalitis patients. mRS scores, ranging from 0 to 5, both on admission and at discharge from the hospital were retrieved by the treating neurologists from clinical charts during hospitalization. Neurologic outcome was dichotomized, with good neurologic outcome defined as mRS scores of 0-3 for patients with no disability to moderate disability at hospital discharge, and poor neurological outcome defined as mRS scores of 4-5 (9).

### 2.4 Statistical Analysis

None of the continuous variables in this study showed a normal distribution, so all data are expressed as medians with interquartile ranges (IQRs) [25% to 75%]. Differences between continuous variables were assessed by the Mann-Whitney *U* test. Categorical variables were compared using χ^2^ test, χ^2^ analysis with continuity correction or Fisher’s exact test, as appropriate. A two-sided P-value of less than 0.05 was considered statistically significant. All statistical analyses were carried out using SPSS, version 25.0, and R, version 3.5.0, for Mac.

### 2.5 Core Variable Selection and Establishment of a Model

The patients in the IVIG + IVMP group were divided into a derivation cohort. In this derivation cohort, least absolute shrinkage and selection operator (LASSO) regression analysis, which is an important method for decreasing the regression coefficient for each variable within a specific range, independent of statistical significance, was performed to select the core variables (10). Possible clinical indicators, including sex, age, and 77 laboratory indicators, were included in the LASSO analysis. This method identified variables that were more representative of the incidence of infection that allowed the identification of an optimally refined generalized linear model without overfitting, which was better suited for the variable analysis of studies with small sample sizes (11). Then, a predictive model was constructed by incorporating the representative variables selected by LASSO into logistic regression. The diagnostic performance of the equation was assessed using receiver operating characteristic (ROC) analysis and then by comparing the area under the curve (AUC), an optimal model was generated. The shiny R Package was used to build an interactive web application. The steps described previously were accomplished using R, version 3.5.0, for Mac.

## 3 Results

### 3.1 Demographic and Clinical Features

From October 2011 to August 2020, we enrolled 278 patients in the West China Hospital, the Third Hospital of Mianyang and Mianyang Central Hospital.

Thirty-four patients (median age: 28 years; female/male: 13:21) received IVMP alone for anti-NMDAR encephalitis. Eighty-four patients (median age: 24 years; female/male: 38:46) received IVIG treatment alone. Additionally, 160 patients (median age: 26 years; female/male: 72:88) received IVIG add to IVMP treatment (IVMP followed IVIG: 44; IVIG followed IVMP: 77; IVIG and IVMP at the same time: 24). The patients with IVMP alone were excluded from statistic comparison for unmatched small size of samples. There were no significant differences in age, sex, or clinical presentations between the two groups. The demographic and clinical features of the patients are summarized in **Table 1**.

**Table 1:** Clinical characteristics of anti-NMDAR encephalitis patients in both groups before immunotherapy.

Variable	IVIG (n = 84)	IVIG + IVMP (n = 160)	*P* value
**Age, years, median (IQR)**	24 (19, 37)	26 (19, 38)	0.467^a^
**Sex, n (%), male**	38 (45.24)	72 (45)	0.972^b^
**Tumors, n (%)**	7 (8.33)	19 (11.88)	0.394^b^
**Cumulative symptoms, n (%)**			
Seizure	58 (69.05)	111 (69.38)	0.958^b^
Behavior dysfunction or cognitive deficits	53 (63.10)	104 (65)	0.768^b^
Disturbance of consciousness	37 (44.05)	89 (55.63)	0.086^b^
Autonomic dysfunction	16 (19.05)	37 (23.13)	0.463^b^
Sleep disturbance	39 (46.43)	71 (44.38)	0.759^b^
**Ancillary examination, n (%)**			
MRI abnormality	30 (35.71)	61 (38.13)	0.711^b^
EEG abnormality	45 (53.57)	90 (56.25)	0.689^b^
CSF protein (> 0.45 g/L)	17 (20.24)	39 (24.38)	0.465^b^
CSF WBC (> 8 cells/μL)	42 (50)	87 (54.38)	0.515^b^

a Mann-Whitney U test.

b Pearson’s χ^2^ test.

### 3.2 In-Hospital Infection Features

**Figure 1:**
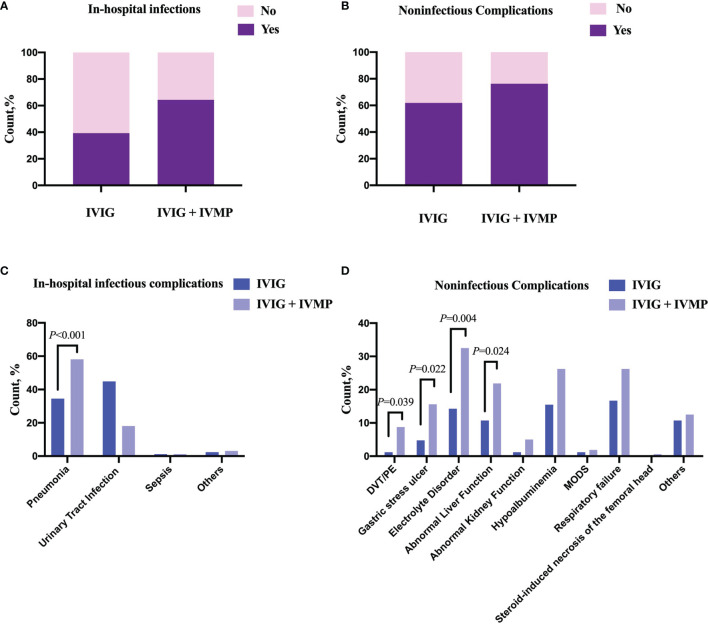
Comparison of the occurrence of complications between anti-NMDAR encephalitis patients with and without large-dose steroids treatment. **(A, B)** The occurrence of infections or noninfectious complications in both groups. Patients in the IVIG + IVMP group had a higher occurrence of infections or noninfectious complications than those in the IVIG group. **(C, D)** Types of complications in both groups. The frequency of pneumonia was highest in the infections followed by the urinary tract infections and sepsis. The proportions of DVT/PE, gastric stress ulcers, electrolyte disorders and abnormal liver function were significantly higher in the IVIG + IVMP group (8.75% vs 1.19%, *P* = 0.039; 15.63% vs 4.76%, *P* = 0.022; 32.50% vs 14.29%, *P* =0.004; 21.88% vs 10.71%, *P* = 0.024, respectively). DVT/PE, deep venous thrombosis/pulmonary embolism; MODS, multiple organ dysfunction syndrome. Differences between variables were compared using χ^2^ test, χ^2^ analysis with continuity correction or Fisher’s exact test, as appropriate.

Our data showed that 103 of 160 patients in the IVIG + IVMP group had one or more episodes of clinical infection during their hospital stay compared with 33 of 84 patients in the IVIG group (**Table 2**). The proportion of infection was significantly higher in the IVIG + IVMP group (*P* < 0.001, **Figure 1A**). These infections included pneumonia in 122 patients, urinary tract infections in 40 patients, sepsis in 3 patients, and other infections (skin infections, acute perinephritis, etc.) in 7 patients (**Figure 1C**).

**Table 2:** Characteristics of complications and outcomes of anti-NMDAR encephalitis patients in both groups.

Variable	IVIG (n = 84)	IVIG + IVMP (n = 160)	*P* value
**No. of infections, n (%)**	33 (39.29)	103 (64.38)	**<0.001** ^a^
Pneumonia	29 (34.52)	93 (58.13)	**<0.001** ^a^
Common	16 (55.17)	48 (51.61)	0.738^a^
Severe	13 (44.83)	45 (48.39)	
Urinary tract infection	11 (13.10)	29 (18.13)	0.313^a^
Sepsis	1 (1.19)	2 (1.25)	0.563^b^
Others	2 (2.38)	5 (3.13)	0.942^c^
**Fever, n (%)**	45 (53.57)	95 (59.38)	0.384^a^
**Duration of fever, median (IQR), days**	4 (3, 13)	7 (4, 13)	0.262^c^
**Body temperature, median (IQR)(°C)**	37.08 (36.72, 37.54)	37.17 (36.88, 37.58)	0.098^d^
<37.5	44 (52.38)	78 (48.75)	0.647^c^
37.5-38.4	32 (38.10)	65 (40.62)	
38.5-39	7 (8.33)	11 (6.88)	
>39	1 (1.19)	6 (3.75)	
**Lung CT/Chest X-ray results, n (%)**	30 (35.71)	96 (60.00)	**<0.001** ^a^
Treatment			
NO. of users of antibiotic, n (%)	35 (41.67)	102 (63.75)	**<0.001** ^a^
NO. of antibiotic, n (%)			
1	20 (57.14)	54 (52.94)	0.908^a^
2	7 (20)	23 (22.55)	
≥3	8 (22.86)	25 (24.51)	
Category of antibiotic, n (%)			
1	21 (60.0)	55 (53.92)	0.817^a^
2	8 (22.86)	26 (25.49)	
≥3	6 (17.14)	21 (20.59)	
Length of use, median (IQR), days	15 (5, 111)	11 (0, 21)	
**Noninfectious Complications**			
NO. of patients of noninfectious complications	52 (61.90)	122 (76.25)	**0.018** ^a^
DVT/PE	1 (1.19)	14 (8.75)	**0.039** ^c^
Gastric stress ulcer	4 (4.76)	25 (15.63)	**0.022** ^c^
Electrolyte disorder	12 (14.29)	52 (32.50)	**0.004** ^a^
Abnormal liver function	9 (10.71)	36 (21.88)	**0.024** ^a^
Abnormal kidney function	1 (1.19)	8 (5.00)	0.253^c^
Hypoalbuminemia	13 (15.48)	42 (26.25)	0.082^a^
MODS	1(1.19)	3 (1.88)	0.896^b^
Respiratory failure	14 (16.67)	42 (26.25)	0.091^a^
Steroid-induced necrosis of the femoral head	0	1 (0.63)	–
Others	9 (10.71)	20 (12.5)	0.682^a^
**CU admission rate, n (%)**	10 (11.90)	17 (10.63)	0.762^a^
**Mortality rate, n (%)**	0	2 (1.25)	–
**LOS, median (IQR), days**	18 (12, 27)	25 (18, 34)	**<0.001** ^d^
**mRS score, median (IQR)**			
Admission	4 (3,5)	4 (3, 5)	0.378^d^
Discharge	2 (1, 3)	3 (2, 5)	**<0.001** ^d^
≤3, n, %	66 (78.57)	90 (56.25)	**<0.001** ^a^
≥4, n, %	18 (21.43)	68 (42.5)	

Bold entries indicate P < 0.05.

DVT/PE, deep venous thrombosis/pulmonary embolism; MODS, multiple organ dysfunction syndrome; LOS, Length of hospital stay.

a Pearson’s χ^2^ test.

b Fisher’s Exact Test.

c Chi-squared test with continuity correction.

d Mann-Whitney U test.

**Figure 2:**
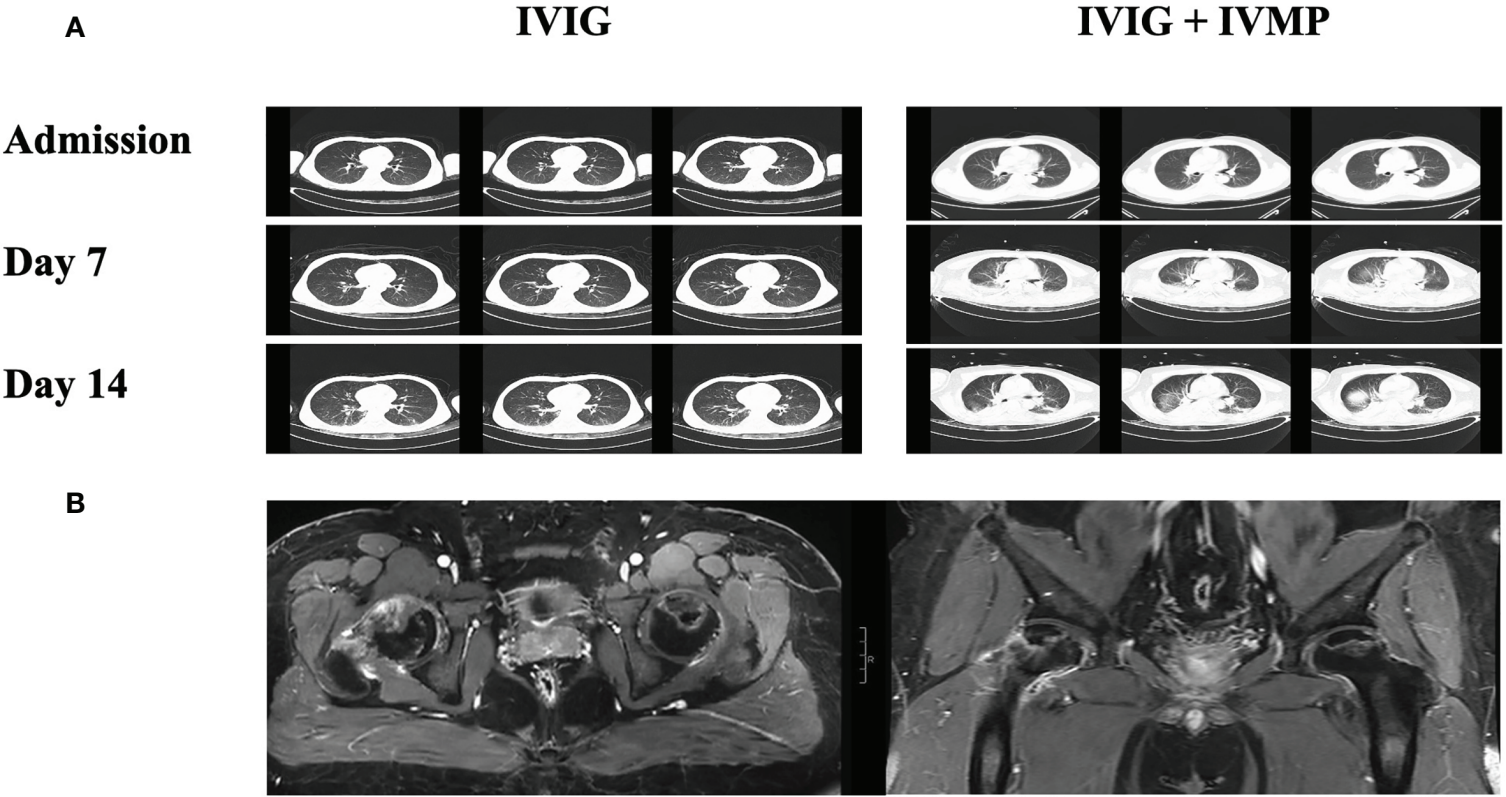
Images of chest CT scan evolution of cases in two groups during treatment and images of steroid-induced necrosis. **(A)** Images of chest CT scan at admission, 7 and 14 days after immunotherapy from cases in IVIG groups and IVIG + IVMP group. **(B)** Images of MRI scan of steroid-induced necrosis from case in IVIG + IVMP group.

Abnormalities in Lung CT or Chest X-ray were found to be more in IVIG + IVMP group (60% vs 35.71%, *P* < 0.001). Images of chest CT scan evolution of cases in two groups during treatment were showed in **Figure 2A**. No significant difference in median body temperature was observed between the two groups within 5 days after first-line immunotherapy (*P* = 0.098). However, the duration of fever was shorter in the IVIG + IVMP group than in the IVIG group, even though there was no significant difference [7 vs. 4; *P* = 0.26].

The monitoring results of inflammatory indicators before and after first-line immunotherapy are shown in **Table 3**. The results of inflammation indicators before immunotherapy were similar in the two groups, except that the WBC count in the IVIG group was higher than that in the IVIG + IVMP group (9.44 vs 7.20, *P* < 0.001). Compared with the IVIG group, WBC count, NLR and SII in the IVIG + IVMP group showed a remarkable increase after treatment (9.93 vs 5.65; 6.94 vs 3.47; 1.47 vs 0.70, respectively, *P* < 0.001). Moreover, lymphocyte-to-monocyte ratio (LMR) was significantly lower (2.20 vs 2.54, *P* = 0.047) in the IVIG + IVMP group. The proportion of patients who received one or more antimicrobial treatments was significantly higher in the IVIG + IVMP group than in the IVIG group (63.75% vs 41.67%, *P* < 0.001).

**Table 3:** Comparisons of laboratory parameters before and after immunotherapy.

Variable	IVIG (n = 84)		IVIG + IVMP (n = 160)		*P* value (IVIG vs. IVIG + IVMP)	
	Before	After	Before	After	Before	After
**WBC, median (IQR) (× 10^9^/L)**	9.44 (6.44, 12.78)	5.65 (4.72, 7.65)	7.20 (5.61, 9.20)	9.93 (7.55, 13.1)	**<0.001**	**<0.001**
**NLR, median (IQR)**	5.08 (3.06, 8.39)	3.47 (2.09, 4.53)	4.05 (2.59, 6.29)	6.94 (3.81, 11.33)	0.068	**<0.001**
**LMR, median (IQR)**	2.71 (1.85, 4.13)	2.54 (1.78, 3.93)	2.87 (1.97, 4.08)	2.20 (1.47, 3.60)	0.684	**0.047**
**SII, median (IQR)**	1.08 (0.63, 1.86)	0.70 (0.44, 1.17)	0.92 (0.54, 1.37)	1.47 (0.84, 2.53)	0.098	**<0.001**
**CRP, median (IQR)(mg/dL)**	4.27 (1.36, 14.6)	7.87 (4.99, 62.78)	4.95 (2.25, 20.00)	8.38 (2.73, 24.40)	0.204	0.359

Bold entries indicate P < 0.05.

WBC, white cell count; NLR, neutrophil-to-lymphocyte ratio in peripheral blood; LMR, lymphocyte-to-monocyte ratio; SII, systemic immune-inflammation index; CRP, C-reactive protein.

Differences between variables were assessed by the Mann-Whitney U test.

The features of pneumonia (the most common infectious complication) and laboratory parameters before and after therapy were analyzed. Body temperature and duration of fever were not different between the two groups. The symptoms/radiologic/laboratory data for pneumonia were list in **Table 4**.

**Table 4:** Symptoms/radiologic/laboratory data for patients with pneumonia in both groups.

Variable	IVIG (n = 29)	IVIG +IVMP (n = 93)	*P* value
**Purulent sputum**	20 (68.97)	68 (73.12)	0.663^a^
**Fever (>38°C)**	26 (89.66)	72 (77.42)	0.238^a^
**Lung CT/Chest X-ray**	29 (100.00)	93 (100.00)	–
**WBC (>10,000 WBC/mm^3^)**	7 (24.14)	45 (48.39)	**0.021** ^a^
**WBC (<4000 WBC/mm^3^)**	6 (20.69)	10 (10.75)	0.16^a^
**Sputum culture**			
G+	1/11 (9.09)	3/33 (9.09)	0.48^b^
G-	8/11 (72.73)	28/33 (84.85)	
Fungus	2/11 (18.18)	2/33 (6.06)	

Bold entries indicate P < 0.05.

WBC, white cell count; G+, Gram-positive bacteria; G-, Gram-negative bacteria.

a Pearson’s χ^2^ test.

b Fisher’s Exact Test.

Clinical infection occurred in 13 patients (37.14%) who received IVMP alone, including pneumonia in 11 patients, urinary tract infections in 1 patient and sepsis in 1 patient. Among them, patients received antimicrobial treatments with a median treatment duration of 17 days. WBC count, NLR and SII in patients received IVMP alone showed a remarkable increase after treatment (10.03 vs 7.01, *P* < 0.05; 6.59 vs 3.48, *P* < 0.001; 1579 vs 890.5, *P* < 0.05).

### 3.3 Comparison of Noninfectious Complications and Outcomes of Anti-NMDAR Encephalitis

Of the 244 patients, 174 (71.31%) had at least one noninfectious complication, including deep venous thrombosis/pulmonary embolism (DVT/PE), gastric stress ulcers, electrolyte disorders, abnormal liver function, abnormal kidney function, hypoalbuminemia, multiple organ dysfunction syndrome (MODS), respiratory failure, steroid-induced necrosis of the femoral head, and other complications (**Figure 1B**). Patients in the IVIG + IVMP group had a significantly higher number of complications than those in the IVIG group (76.25% vs 61.90%, *P* = 0.018; **Figure 1B**). The proportions of DVT/PE, gastric stress ulcers, electrolyte disorders and abnormal liver function were significantly higher in the IVIG + IVMP group (8.75% vs 1.19%, *P* = 0.039; 15.63% vs 4.76%, *P* = 0.022; 32.50% vs 14.29%, *P* = 0.004; 21.88% vs 10.71%, *P* = 0.024, respectively; **Figure 1D**). Images of MRI scan of steroid-induced necrosis from case in IVIG + IVMP group were showed in **Figure 2B**.

LOS was significantly longer in the IVIG + IVMP group than in the IVIG group (25 vs 18, *P* < 0.001). There was no difference in ICU stay or mortality between the two groups.

The two groups had similar mRS scores on admission (4, *P* = 0.378). However, the IVIG group had a lower mRS score than the IVIG + IVMP group at discharge from the hospital (2 vs. 3, *P <*0.001) Additionally, the percentage of patients in the IVIG group in the good prognosis was higher at the time of discharge (78.57% vs 56.25%, *P* < 0.001; **Supplemental Figure 1**).

In patients with IVMP alone, the mRS scores at discharge from the hospital were higher than admission (2 vs. 3, *P <*0.05) and 10 (29.41%) patients in the worse prognosis at the time of discharge.

### 3.4 Core Variable Selection and Establishment of the Predictive Model

**Figure 3:**
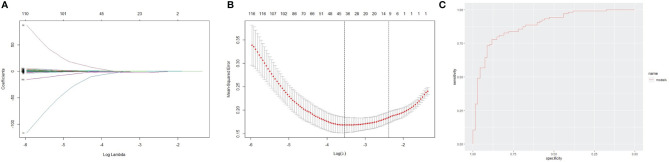
Lasso feature selection diagram and receiver operator characteristic curve. **(A)** Coefficient diagram of least absolute shrinkage and selection operator (LASSO) variables. Each curve in the figure represents the trajectory of the coefficient of an independent variable. The x-coordinate is the logarithm function of the penalty coefficient λ, and the y-coordinate is the mean square error. As lambda changes, the coefficient of the variable is compressed to zero. **(B)** Adjustment parameters in the LASSO model; The dotted line on the left represents the value of the λ log function with the minimum mean squared error, and the right represents the best lambda log function. **(C)** Receiver operator characteristic curve. The AUC was 0.885 for the training cohort.

We investigated the possibility of identifying high dose steroid-induced in-hospital infection patients based on candidate variables. Using LASSO regression analysis for multivariate analysis, seven core variables (mRS score on admission, seizure, body temperature, uric acid (URIC), CSF IgG, NLR and LMR) were selected out of 89 possible indicators before immunotherapy to formulate a disease panel (**Figures 3A, B**). The core variables were integrated into a LASSO model and assigned weighting coefficients. Afterward, the seven core variables were combined to obtain a scoring formula. The ROC curve was generated to validate the predictive accuracy of the model. The ROC curve showed an AUC of 0.885 in the training set, revealing that the model had good reliability (**Figure 3C**).

## 4 Discussion

Patients with anti-NMDAR encephalitis frequently present susceptibility to various etiological infections due to abnormal immunity; however, few studies have reported side effects that lead to infection of high dose steroids as first-line immunotherapy. In this retrospective-multiple-centers study, almost two thirds of patients treated with IVIG and steroid therapy had infectious events, apparently higher than those who received IVIG therapy alone (39%). In our study, pneumonia was the most common infection, and nearly half of the patients treated with high dose steroids presented serious pneumonia and accepted ventilator assistance for peripheral respiratory failure. The finding revealed the effect of high dose steroids on immunosuppression and promoting in-hospital infection. The role of high dose steroid as first-line treatment may be reconsidered.

In this study, we also observed that patients treated with high dose steroid therapy had higher proinflammatory laboratory parameters and used antibiotics more. Compared to inflammatory symptoms and physical signs, inflammation-related indicators are more sensitive. Before immune treatment, NLR, LMR and SII were not different between high dose steroids and IVIG groups. However, WBC count, NLR and SII significantly increased after therapy in patients with high dose steroid. Moreover, LMR was also significantly lower after therapy in these patients. The Increased WBC count is a common side effect of steroids (12, 13). NLR is considered an important marker of the systemic inflammatory response and objectively reflects the degree of the systemic inflammatory response, the severity of the inflammatory response and the immune status of the body (14). Activated NLR is frequently associated with poor outcome in inflammatory diseases or secondary infection after cancer (15, 16). Similar to NLR, SII is a marker related to inflammatory status (17–20). Some studies have shown that increased NLR or SII is associated with poor outcomes in patients with cancer or cardiovascular and cerebrovascular disease and those who have secondary infection (21–25). A decreased LMR is associated with infection in liver cirrhosis and an adverse prognosis in cancer (26). This is consistent with the result of our study.

Several retrospective studies have shown that early and aggressive steroid therapy is associated with good outcomes in anti-NMDAR encephalitis patients (4, 8). However, our data showed that patients who received high dose steroid therapy had longer in-hospital stays and poorer short-term outcomes than those who received IVIG therapy. This may be associated with a higher occurrence of noninfectious complications, such as DVT/PE, gastrointestinal bleeding, abnormal liver function, and abnormal kidney function, in patients with anti-NMDAR encephalitis treated with high dose steroid therapy than in patients treated with IVIG therapy. In addition, one patient had steroid-induced necrosis of the femoral head. In the present study, among three patients who died, two patients in the steroid group died due to multiple organ failure. Previous studies also reported that severe complications were the main causes of death, and the use of steroids contributed to significantly more aggravated or induced infections and poorer short-term outcomes (6, 27).

The LASSO algorithm identified seven core variables, including mRS score on admission, seizure, body temperature, URIC, CSF IgG, NLR and LMR, which were integrated with a multiparameter combination. The performance evaluation of this model demonstrated that it had good reliability and accuracy, with a satisfactory AUC of 0.885 in the training set. The prediction model theoretically and statistically displayed a certain correlation between laboratory or clinical variables and infectious complications, which provides clues for the selection of treatment options regarding whether to use high dose steroids. High mRS scores on admission and frequent seizures were indicators of illness severity. Consistent with previous results in our study, the predictive model showed that NLR and LMR was strongly associated with inflammatory status and poorer prognosis. Although there were no differences in URIC, CSF IgG or fever between the two groups, the LASSO model revealed the role of these parameters in predicting infection. A recent study also revealed that fever is an independent risk factor for the exacerbation of anti-NMDAR encephalitis (28, 29). Multiple parameters make better predictions of infection than one parameter alone. This prediction model will help doctors develop reasonable treatment strategies for anti-NMDAR patients (30).

Our study still has several limitations. We did not use a validation cohort for LASSO analysis due to the relatively small sample size of patients treated with high dose steroids. The validation and clinical application of LASSO in the real world are needed in the future. We had ever considered include IVMP group into statistical analysis, however, some factors may influence the results: First, the sample size of the patients received high dose steroids alone was very small, which may have a greater effect on balance among three groups than the mismatch between the IVIG and IVIG + IVMP groups, and may increase statistical bias. Second, unbalance of sample size among three groups may be attributed to retrospective but not prospective study. Finally, lower mRS score on admission (3 vs 4 vs 4) and shorter in-hospital stay (17 vs 18 vs 25) was shown in IVMP group than IVIG group and IVIG + IVMP group (**Supplemental Table 1**). It reminded that conditions were not serious in IVMP group. In real world, according to doctor’s experience, IVIG or IVIG + IVMP may be chosen as treatment strategies more frequently when patient was in serious conditions. The above factors may reduce the efficacy of the statistical test. It should be recognized as such that steroids therapy is truly two sides of the same coin for the immune benefit it brings and its main side effect on worsening infection. It should be recognized as such that steroids therapy is truly two sides of the same coin for the immune benefit it brings and its main side effect on worsening infection. The findings underscore the need of individualized immunotherapy strategy for patients with different conditions. We will enroll more patients with anti-NMDAR encephalitis in the future to expand the sample size for verification.

## 5 Conclusions

In conclusion, high dose steroids therapy was significantly associated with higher in-hospital infectious complication rates and a poor short-term prognosis in relatively severe anti-NMDAR encephalitis patients. The prediction model that included mRS score on admission, seizure, body temperature, URIC, CSF IgG, NLR and LMR might aid in the selection of first-line immunotherapy to reduce the risk of in-hospital infection clinically.

## Data Availability Statement

The datasets analyzed in this article are anonymous to protect patient privacy and are not publicly available. Request to access the datasets should be directed to email the corresponding authors.

## Ethics Statement

The studies involving human participants were reviewed and approved by Research Ethics Committee of the Medical School of Sichuan University. The patients/participants provided their written informed consent to participate in this study. Written informed consent was obtained from the individual(s) for the publication of any potentially identifiable images or data included in this article.

## Author Contributions

JW, JFL, and MW carried out the statistical analysis and drafted the manuscript. ZM collected and interpreted the data. DZ and JML conceptualized and designed the study and revised the manuscript. All authors contributed to the article and approved the submitted version.

## Funding

This work was supported by the National Natural Science Foundation of China (Grant No. 81571272 and 82071459) and Sichuan province science and technology project (Grant No. 2019YFH0145).

## Conflict of Interest

The authors declare that the research was conducted in the absence of any commercial or financial relationships that could be construed as a potential conflict of interest.

## Publisher’s Note

All claims expressed in this article are solely those of the authors and do not necessarily represent those of their affiliated organizations, or those of the publisher, the editors and the reviewers. Any product that may be evaluated in this article, or claim that may be made by its manufacturer, is not guaranteed or endorsed by the publisher.

## Supplementary Material

Supplementary Figure 1 Neurologic outcome of the study population. The figure demonstrates percent distribution of Modified Rankin Scale (mRS) score profiles patients in both groups at **(A)** admission to the hospital and **(B)** discharge from the hospital.
