# Supplementary figures and images for: High Dose Steroids as First-Line Treatment Increased the Risk of In-Hospital Infections in Patients With Anti-NMDAR Encephalitis
Source: Front Immunol. 2021 Dec 17;12:774664. doi: 10.3389/fimmu.2021.774664 (PMC8718407; doi:10.3389/fimmu.2021.774664)

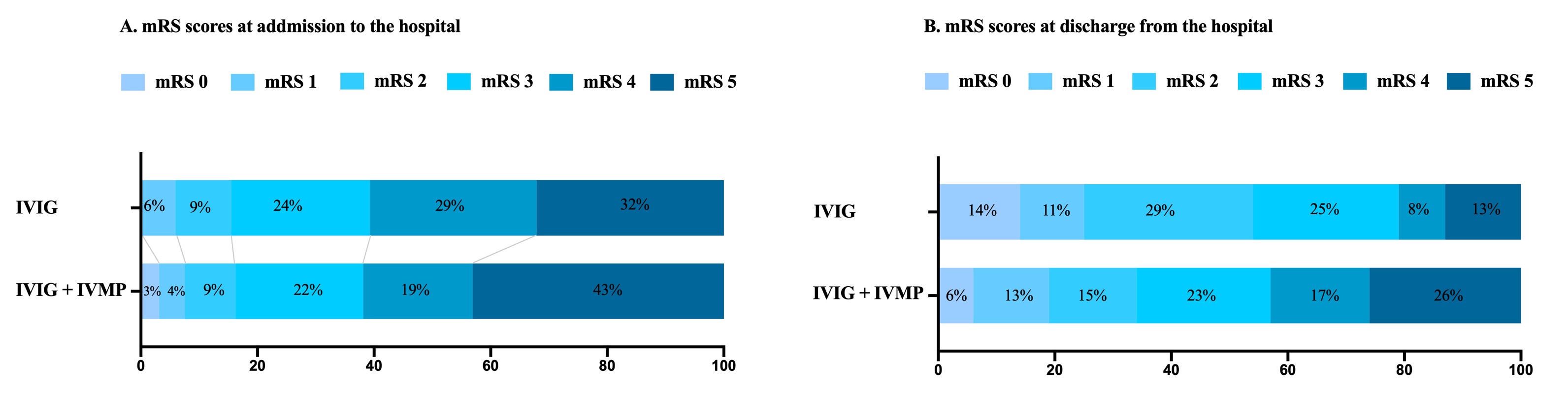

Supplement: Supplementary Figure 1 — Neurologic outcome of the study population. The figure demonstrates percent distribution of Modified Rankin Scale (mRS) score profiles patients in both groups at (A) admission to the hospital and (B) discharge from the hospital. [file Image_1.jpeg]
