# Supplementary material for: High Dose Steroids as First-Line Treatment Increased the Risk of In-Hospital Infections in Patients With Anti-NMDAR Encephalitis
Source: Front Immunol. 2021 Dec 17;12:774664. doi: 10.3389/fimmu.2021.774664 (PMC8718407; doi:10.3389/fimmu.2021.774664)
Supplement: Supplementary file 2 [file Table_1.pdf]

Table 1 Characteristics of complications and outcomes of anti-NMDAR encephalitis patients in both groups

Variable	IVMP (n=34)	IVIG (n=84)	IVIG + IVMP (n=160)	*P* value	Post hoc
**No. of infections, n (%)**	13 (37.14)	33 (39.29)	103 (64.38)	**< 0.001**[a**]	**G3 > G1, G3 > G2**[a**]
Pneumonia	11 (32.35)	29 (34.52)	93 (58.13)	**< 0.001**[a**] 0.937[a]	**G3 > G1, G3 > G2**[a**]
Common	6 (54.54)	16 (55.17)	48 (51.61)		
Severe	5 (45.46)	13 (44.83)	45 (48.39)		
Urinary tract infection	1 (2.94)	11 (13.10)	29 (18.13)	0.067[a]	
Sepsis	1 (2.94)	1 (1.19)	2 (1.25)	-	
Others	1 (2.94)	2 (2.38)	5 (3.13)	-	
**Fever, n (%)**	13 (38.23)	45 (53.57)	95 (59.38)	0.076[a]	
**Duration of fever, median (IQR), days**	4 (1, 8)	4 (3, 13)	7 (4, 13)	0.199[b]	
**Body temperature, median (IQR)(°C)**	36.76 (36.61, 37.11)	37.08 (36.72, 37.54)	37.17 (36.88, 37.58)	**< 0.001**[b**]	**G3 > G1, G2 > G1**[b**]
<37.5	24 (70.59)	44 (52.38)	78 (48.75)		
37.5-38.4	9 (26.47)	32 (38.10)	65 (40.62)		
38.5-39	1 (2.94)	7 (8.33)	11 (6.88)		
>39	0	1 (1.19)	6 (3.75)		
**Lung CT/ Chest X-ray results, n (%)**	10 (29.41)	30 (35.71)	96 (60.00)	**< 0.001**[a**]	**G3 > G1, G3 > G2**[a**]
**Treatment**					
NO. of users of antibiotic, n (%)	11 (32.35)	35 (41.67)	102 (63.75)	**< 0.001**[a**]	**G3 > G1, G3 > G2**[a**]
NO. of antibiotic, n (%)					
1	5 (45.45)	20 (57.14)	54 (52.94)	0.967[c]	
2	3 (27.27)	7 (20)	23 (22.55)		
≥3	3 (27.27)	8 (22.86)	25 (24.51)		
Category of antibiotic, n (%)					
1	5 (45.45)	21 (60.0)	55 (53.92)	0.661[a]	
2	5 (45.45)	8 (22.86)	26 (25.49)		
≥3	1 (9.09)	6 (17.14)	21 (20.59)		
Length of use, median (IQR), days	17 (7, 23)	15 (11, 28)	16 (11, 30)	0.885[b]	
**Noninfectious Complications**					
NO. of patients of noninfectious complications	19 (55.88)	52 (61.90)	122 (76.25)	**0.013**[a*]	**G3 > G1, G3 > G2**[a*]
DVT/PE	2 (5.88)	1 (1.19)	14 (8.75)	0.064[c]	

Gastric stress ulcer	3 (8.82)	4 (4.76)	25 (15.63)	**0.036**[c*]	**G3 > G2**[c*]
Electrolyte disorder	7 (20.59)	12 (14.29)	52 (32.50)	**0.006**[a**]	**G3 > G2**[a*]
Abnormal liver function	5 (14.71)	9 (10.71)	36 (21.88)	0.065[a]	
Abnormal kidney function	1 (2.94)	1 (1.19)	8 (5.00)	0.325[c]	
Hypoalbuminemia	2 (5.88)	13 (15.48)	42 (26.25)	**0.011**[a*]	**G3 > G1**[a*]
MODS	0	1(1.19)	3 (1.88)	-	
Respiratory failure	5 (14.71)	14 (16.67)	42 (26.25)	0.126[a]	
Steroid-induced necrosis of the femoral head	1 (2.94)	0	1 (0.63)	-	
Others	0	9 (10.71)	20 (12.5)	-	
**ICU admission rate, n (%)**	0	10 (11.90)	17 (10.63)	-	
**Mortality rate, n (%)**	1 (2.94)	0	2 (1.25)	-	
**LOS, median (IQR), days**	17 (12, 23)	18 (12, 27)	25 (18, 34)	**< 0.001**[b**]	**G3 > G1, G3 > G2**[b**]
**mRS score, median (IQR)**					
Admission	3 (2, 5)	4 (3,5)	4 (3, 5)	0.025[b*]	**G2 > G1, G3 > G3**[b*]
Discharge	1 (1, 4)	2 (1, 3)	3 (2, 5)	**< 0.001**[b**]	**G3 > G1, G3 > G2**[b**]
≤ 3, n, %	23 (67.65)	66 (78.57)	90 (56.25)	**0.003**[a**]	**G3 > G2**[a**]
≥ 4, n, %	10 (38.24)	18 (21.43)	68 (42.5)		

Bold entries indicate $P < 0.05$.
Abbreviations: DVT/PE = deep venous thrombosis/pulmonary embolism; MODS = multiple organ dysfunction syndrome; LOS = Length of hospital stay; G1 = IVMP group; G2 = IVIG group; G3 = IVIG + IVMP group.
*$P < 0.05$ and**$P < 0.01$
[a] Pearson's χ^2 test.
[b] Mann-Whitney *U* test.
[c] Fisher's Exact Test.
